# Associations of rDNA copy numbers and global DNA methylation with myocardial infarction

**DOI:** 10.3389/fcvm.2026.1740048

**Published:** 2026-05-04

**Authors:** Xiao Wang, Ashfaque A. Memon, Anna Hedelius, Anton Grundberg, Kristina Sundquist

**Affiliations:** 1 Center for Primary Health Care Research, Department of Clinical Sciences Malmö, Lund University, Malmö, Sweden; 2 University Clinic Primary Care, Skåne University Hospital, Malmö, Region Skåne, Sweden

**Keywords:** cardiovascular disease, epigenetics, global DNA methylation, myocardial infarction, rDNA copy number

## Abstract

**Background:**

Alterations in ribosomal DNA copy number (rDNA-CN) and global DNA methylation have been associated with genomic instability and various diseases. However, their relevance in myocardial infarction (MI) has not been fully investigated.

**Methods:**

We measured rDNA-CN and global DNA methylation in blood samples from MI patients (*n* = 100) and healthy controls (*n* = 109) using Droplet Digital PCR (ddPCR) and ELISA, respectively. Logistic regression was used to assess associations with MI, before and after adjustment for age and sex. Correlations of rDNA CN and global DNA methylation with age were also evaluated. Discriminatory performance was assessed using receiver operating characteristic (ROC) curve analysis.

**Results:**

rDNA-CN was associated with MI in the unadjusted analysis (OR=0.991, *p* < 0.001), but the effect was no longer significant after adjusting for age and sex (OR=0.992, *p* = 0.06). In contrast, global DNA methylation remained significantly associated with MI after adjustment (OR=1.61, *p* < 0.001). Global DNA methylation also demonstrated strong discriminative ability for MI (AUC = 0.97).

**Conclusions:**

Global DNA methylation was independently associated with MI after the adjustments, whereas rDNA-CN was not. These findings suggest that global DNA methylation may represent a potential biomarker; however, further validation in larger, well-characterized populations is warranted.

## Introduction

Ribosomal DNA (rDNA) encodes the 18S, 5.8S, and 28S ribosomal RNA (rRNA) subunits, which are essential for ribosome biogenesis; this in turn is necessary for protein synthesis in eukaryotic cells (1). In humans, rDNA is organized in multiple tandem repeats within nucleolar organizer regions of specific chromosomes (2). Although only 30%–50% of rDNA copies are transcriptionally active, the total copy number (CN) influences ribosome production, nucleolar function, and genomic stability (3–6).

rDNA copy number (rDNA-CN) varies greatly among individuals and is increasingly recognized as a dynamic genomic feature. Variability in rDNA-CN has been linked to stress response, aging, body mass index (BMI), as well as several diseases, including cancer, neurodegeneration, and impaired renal function (2, 5, 7–14). Moreover, rDNA dosage is regulated by DNA methylation, positioning it at the intersection of genetic and epigenetic regulation (3, 10). Global DNA methylation, which reflects genome-wide methylation predominantly occurring at cytosine–phosphate–guanine (CpG) sites, is a key epigenetic marker associated with cancer, type 2 diabetes, and cardiovascular disease (CVD) (15–19). Blood-based global methylation assays have shown diagnostic potentials, with commercial tests already available for colorectal cancer (16). However, the interplay between rDNA-CN and global methylation, and their combined relevance to CVD, remains largely unexplored.

Myocardial infarction (MI), a leading cause of morbidity and mortality worldwide, arises from complex interactions between genetic, environmental, and epigenetic factors. Ribosome dysfunction and nucleolar stress, triggered by ischemia, contribute to cardiomyocyte injury and maladaptive remodeling (20). Cardiac ischemia can induce nucleolar stress, impacting rRNA transcription and disrupting ribosome biogenesis, which can activate stress response pathways and contribute to cardiomyocyte injury and dysfunction (21). While numerous genetic risk factors for MI have been identified, the role of structural genomic variations such as rDNA-CN remains underexplored. Given the links between rDNA dosage, stress adaptation, and genomic stability, rDNA-CN may be relevant to MI pathogenesis. Global DNA methylation has been associated with CVD, but its interplay with rDNA-CN is unexplored.

In this study, we investigated the associations of rDNA-CN and global DNA methylation with MI and evaluated their discriminative potential. To the best of our knowledge, this is the first study investigating the role of both rDNA-CN and global DNA methylation in MI and their correlations as well as discriminative potential.

## Material and methods

### Study population

A total of 100 MI patients who were diagnosed with MI (ICD10: I21-I22) or ischemic heart disease and/or had undergone balloon angioplasty or coronary artery bypass surgery (CABG), aged 45–80 years, were included in the Södertälje study between 2007 and 2009. All the patients were registered in Salem, Nykvarn or Södertälje and admitted to Södertälje Hospital or Karolinska University Hospital, Huddinge. The control group included 109 healthy volunteers, aged 20–67 years, recruited in 2015 from the Malmö blood center. All participating individuals met the standard Swedish Blood donor criteria for blood donation.

### Blood sampling

Genomic DNA was extracted from 200 μL of frozen whole blood using the Puregene kit for MI samples and the QIAamp 96 DNA Blood Kit (Qiagen, Hilden, Germany) for Blood donor samples. DNA purity was assessed using a NanoDrop spectrophotometer, with OD 260/280 > 1.8 and concentration (Qubit 4.0, Life Technologies) following the manufacturer's protocol. Extracted DNA was stored at −20 °C until further use.

### Quantification of rDNA copy number by droplet digital PCR (ddPCR)

Absolute quantification of rDNA-CN was performed using a well-optimized droplet digital PCR (ddPCR) method (22). Primers and probes targeting the human 28S rDNA and the reference gene eukaryotic translation initiation factor 2C, 1 (EIF2C1; also known as Argonaute 1, Gene ID: 26523), were obtained from Bio-Rad (Hercules, CA, USA). Probes were labeled with FAM for rDNA and HEX for EIF2C1, both quenched with Iowa Black FQ. For rDNA: the primer and probe sequences for rDNA were: Forward Primer 5’- TGATGTGTTGTTGCCATGGT-3’; Reverse primer 5’-AGCCAAGCACATACACCAAA-3’; Probe 5’- AGTACGAGAGGAACCGCAGG-3’. For EIF2C1, the assay (ID: dHsaCP1000002, sequence accession number: NM_012199.2) included the following context sequence: TGGTTCGGCTTTCACCAGTCTGTGCGCCCTGCCATGTGGAAGATG ATGCTCAACATTGATGGTGAGTGGGGAGAGCTATGGAGCCAGGG GCACCCCAAGTCCAGTGACCACACTCCCAGCCTC.

Each ddPCR reaction (20 μL) contained 2 ng of purified genomic DNA, 900 nM of each primer and probe, 1× ddPCR Supermix for Probes (No dUTP; Bio-Rad), and 5 U/reaction HaeIII restriction enzyme (Thermo Scientific, Hudson, NH, USA). The reaction mixture was incubated at room temperature for 20 min to ensure DNA digestion, after which droplets were generated using the Automated Droplet Generator (Bio-Rad).

PCR conditions were set as follows: 95 °C for 10 min (enzyme activation), followed by 40 cycles of 94 °C for 30 s (denaturation) and 60 °C for 1 min (annealing/extension), and 98 °C for 10 min (enzyme deactivation). PCR plates were incubated overnight at 4 °C to enhance droplet stability, yielding 19,000–20,000 droplets per well.

Droplets were read with a QX200 Droplet Reader (Bio-Rad), and QuantaSoft Software (Bio-Rad) was used to calculate copy numbers based on FAM- and HEX-positive droplets using Poisson statistics. Each run included positive and negative controls.

### Global DNA methylation assessment

Genomic DNAs were diluted to 20 ng/μl. Absolute global 5-methyl cytosine (5-mc) levels were quantified in 100 ng DNA using the MethylFlash™ Global DNA Methylation Quantification Kit (Epigentek) according to the manufacturer's instructions (23). Absorbance was measured at 450 nm (Infinite® F200, Tecan), and methylation levels were calculated from a standard curve (0.1%–5% methylated DNA). Standards were prepared fresh for each plate and were run in duplicates. Samples were analyzed in duplicate, with intra- and inter-assay coefficients of variation (CVs) of less than 10%.

### Statistical analysis

Continuous variables with a normal distribution, including age, rDNA-CN, body mass index (BMI), systolic blood pressure (SBP), diastolic blood pressure (DBP), total cholesterol, high-density lipoprotein (HDL), and low-density lipoprotein (LDL), were summarized as mean ± standard deviation (SD). Non-normally distributed variables, such as global DNA methylation and triglycerides, were expressed as median and interquartile range (IQR). Categorical variables, including sex, smoking status, education, and family history of MI, were presented as percentages. These variables were examined for potential associations with rDNA-CN and global DNA methylation.

Group comparisons were performed using Student's t-test for rDNA-CN and Mann–Whitney U test for global DNA methylation. The correlation between rDNA-CN and age was assessed using Pearson's correlation, while the correlation between global DNA methylation and age were evaluated using Spearman's rank correlation. Correlations were also examined within each group, given that controls were generally younger than MI patients.

Univariate logistic regression was used to estimate odds ratios (ORs) for MI, followed by multivariate models adjusted for age and sex (note: only these two variables were available for the healthy control group). Receiver operating characteristics (ROC) curves were generated to evaluate the discriminatory performance of rDNA-CN and global DNA methylation, with the area under the curve (AUC) reported alongside 95% confidence intervals (CIs), sensitivity, and specificity. Additionally, a sensitivity analysis was performed in which ORs and AUCs were estimated in participants within the same age range in both patients and controls.

A two-tailed *p*-value <0.05 was considered statistically significant. All analyses were performed using IBM SPSS Statistics version 29 (IBM, New York, USA), and R version 4.5.3 (R Core Team, 2026).

## Results

### Population characteristics

Baseline characteristics of the population are presented in Table 1. Patients with MI were older than controls (64.0 ± 8.5 vs. 43.5 ± 12.5 years) and included a higher proportion of males (77% vs. 60%). They had a mean BMI of 23.6, were more often smokers (59%), and more commonly reported ≤12 years of education. A family history of cardiovascular disease was observed in 50% of patients. Mean SBP and DBP were 139 and 84 mmHg, respectively.

**Table 1:** Characteristics of the study population.

Variables	Patients (*n* = 100)	Control subjects (*n* = 109)	*P*-value
Age, years Mean (SD)	64.0 (8.5)	43.5 (12.5)	<0.001^a^
Sex Male/female (%)	77/23	60/40	0.007^b^
rDNA-CN^e^ Mean (SD)	214 (47)	237 (53)	<0.001^c^
Global DNA methylation (%)^f^ Median (IQR)	0.24 (0.08)	0.08 (0.04)	<0.001^d^
BMI Mean (SD)	26.6 (3.4)	N/A	N/A
Smoker, % Yes/no	59/41	N/A	N/A
Education (%)^g^ <12 years/> 12	64/32	N/A	N/A
Family history of MI (%) Yes/No	50/50	N/A	N/A
Systolic blood pressure, mmHg Mean (SD)	139 (18)	N/A	N/A
Diastolic blood pressure, mmHg Mean (SD)	84 (9)	N/A	N/A
Total cholesterol, mmol/L Mean (SD)	4.3 (1.1)	N/A	N/A
Triglycerides, mmol/L Median (IQR)	1.3 (1.1)	N/A	N/A
HDL, mmol/L Mean (SD)	1.1 (0.3)	N/A	N/A
LDL, mmol/L Mean (SD)	2.6 (1.2)	N/A	N/A

SD, standard deviation; IQR, interquartile range; N/A, not available.

a P-value is given for the comparison of patients and control subjects using an independent sample t-test.

b P-value is based on Chi-square test.

c P-value is given for the comparison of patients and control subjects using an independent sample t-test.

d P-value is given for the comparison of patients and control subjects using a Mann–Whitney U test.

e 1 sample in MI group was missing.

f 7 in MI group and 4 in controls were missing.

g 4% had missing values on education.

### rDNA-CN and global DNA methylation

The rDNA-CN was normally distributed and significantly lower in MI patients compared with controls (214 vs. 237, *p* < 0.001, Table 1). In contrast, global DNA methylation was skewed and significantly higher in patients than in controls (0.25% vs. 0.08%, *p* < 0.001, Table 1).

Age was inversely correlated with rDNA-CN and positively correlated with global DNA methylation in the whole study population, but these correlations were not observed after stratification by MI status (Supplementary Figures S1, 2). rDNA-CN and global DNA methylation were inversely correlated overall, but this relationship disappeared after stratification by MI status (Supplementary Figure S3). After adjustment for age and sex, neither rDNA-CN nor global DNA methylation was associated with other variables listed in Table 1, except for a positive association between BMI and rDNA-CN (*β* = 3.02; *p* = 0.04) (Supplementary Tables S1, 2).

### Association between rDNA -CN, global DNA methylation, and MI

In unadjusted analysis, lower rDNA-CN was weakly but significantly associated with MI (OR=0.991, *p* < 0.001); however, this association was further attenuated after adjustment for age and sex and no longer remained significant (OR=0.992, *p* = 0.06) (Table 2). In contrast, global DNA methylation was significantly associated with MI even after adjustment (OR=1.61, *p* < 0.001). The findings were consistent in sensitivity analysis restricted to participants within the same age interval (45–67 years, Table 3). The potential mechanistic links between MI and rDNA-CN and global DNA methylation are illustrated in Supplementary Figure S4.

**Table 2:** Associations between rDNA-CN, global DNA methylation, and MI (*n* = 209).

	Univariate analysis			Multivariate analysis		
Variables	OR	*p*-value	95% CI	OR	*p*-value^b^	95% CI
rDNA-CN	0.991	0.001	0.985; 0.996	0.992	0.064	0.984; 1.000
Global DNA methylation^a^	1.526	<0.001	1.378; 1.740	1. 613	< 0.001	1.383; 2.010

a Global DNA methylation values were multiplied by 100 (expressed as a percentage) for interpretability.

b Logistic regression analysis adjusted for age and sex.

**Table 3:** Associations between rDNA-CN, global DNA methylation, and MI using groups restricted to the same age interval (45–67 years, *n* = 121).

	Univariate analysis			Multivariate analysis		
Variables	OR	*p*-value	95% CI	OR	*p*-value^b^	95% CI
rDNA-CN	0.990	0.015	0.982; 0.998	0.992	0.069	0.984; 1.000
Global DNA methylation^a^	1.495	<0.001	1.323; 1.761	1.576	<0.001	1.355; 1.957

a Global DNA methylation values were multiplied by 100 (expressed as a percentage) for interpretability.

b Logistic regression analysis adjusted for age and sex.

### Discriminative ability

**Figure 1:**
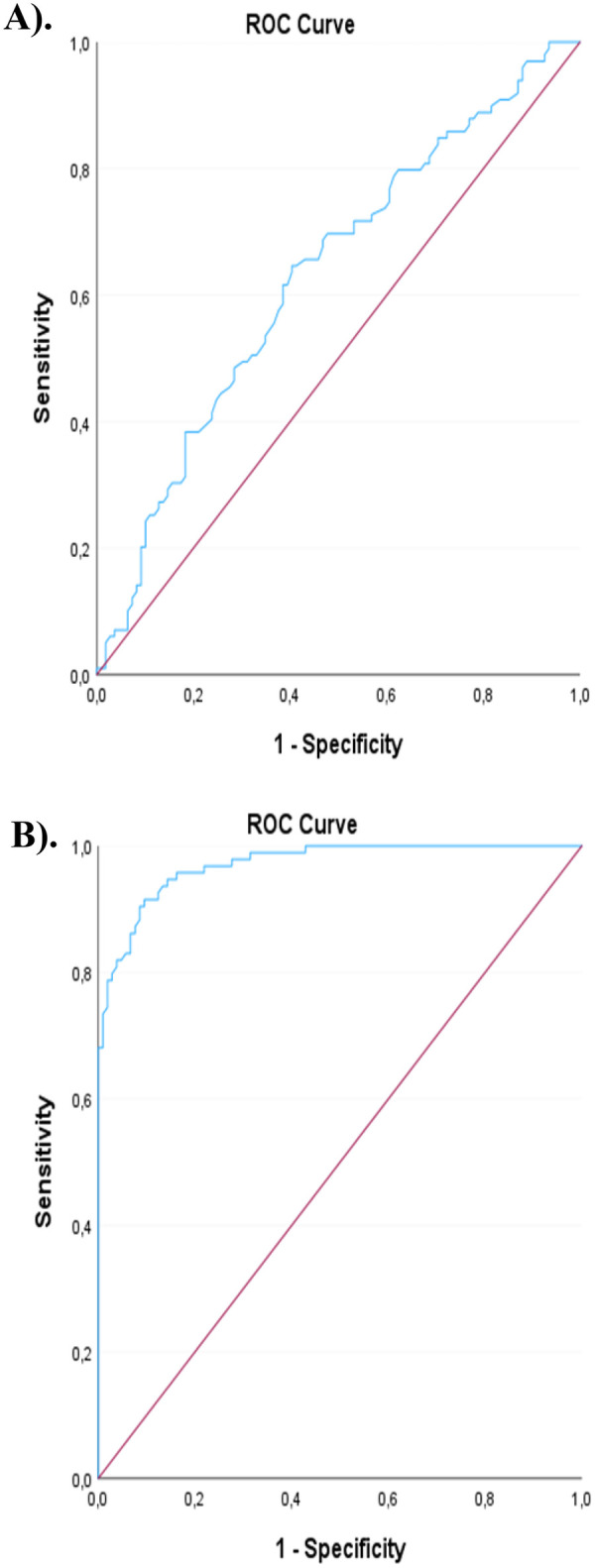
ROC curves showing AUC for: **A**) rDNA-CN and **B**) global DNA methylation.

ROC analysis showed that rDNA-CN had modest discriminative ability for MI (AUC=0.63; Figure 1A), whereas global DNA methylation exhibited strong performance (AUC = 0.97; Figure 1B) with a specificity of 0.90 and sensitivity of 0.91 (Table 4). Similar results were observed in sensitivity analyses restricted to participants within the same age interval (45–67 years) (Table 5).

**Table 4:** AUC for prediction of MI with 95% CI for rDNA-CN and global DNA methylation, along with sensitivity and specificity at the optimal cut-off.

Variables	AUC	*p*-value	95% CI	Sensitivity	Specificity
rDNA-CN	0.63	0.001	0.55; 0.70	0.62	0.62
Global DNA methylation	0.97	<0.001	0.95; 0.99	0.91	0.90

AUC: Area under the curve; CI: confidence intervals.

**Table 5:** AUC (95% CI) for predicting MI using rDNA-CN and global DNA methylation in age-restricted groups (45–67 years, *n* = 121), with corresponding sensitivity and specificity at the optimal cut-off.

Variables	AUC	*p*-value	95% CI	Sensitivity	Specificity
rDNA-CN	0.63	0.006	0.54; 0.73	0.56	0.67
Global DNA methylation	0.97	<0.001	0.94; 0.99	0.89	0.92

AUC, area under the curve; CI, confidence intervals.

## Discussion

In this study, we investigated the associations of rDNA-CN and global DNA methylation with MI and identified three major findings. First, rDNA-CN was lower, albeit with a weak effect size, in patients with MI compared to controls and this association was no longer significant after adjustment for age and sex. Second, global DNA methylation was higher in MI patients and remained independently associated with MI after adjustment, showing strong discriminative ability (AUC = 0.97).

Although no studies have directly investigated rDNA-CN in cardiovascular disease, reduced rDNA-CN has been proposed as a marker of cumulative genomic instability. In this study, the attenuation of the rDNA-CN association after age and sex adjustment highlights the observed relationship may be influenced by confounding factors rather than representing an independent association. In addition, the relatively modest sample size may have limited ability to detect small effects. rDNA arrays are highly dynamic regions of the genome that undergo instability, contributing to altered nucleolar function and cellular stress responses (24, 25). Lower rDNA-CN may reflect reduced ability to adapt to cellular stress, which could be relevant in the context of cardiovascular disease. Our findings suggest that, in this study population, rDNA-CN was not independently associated with MI.

In contrast, global DNA methylation demonstrated a robust and independent association with MI. Increased methylation in blood DNA may reflect systemic epigenetic reprogramming in response to chronic inflammation, oxidative stress, and metabolic dysfunction, all of which are key contributors to atherosclerosis and ischemic heart disease (26–28). For instance, a multi-cohort study involving 2,321 American Indian adults identified 505 differentially methylated positions in blood DNA samples that were associated with incident coronary heart disease (CHD). This suggests that blood DNA methylation can predict CHD risk (29). Similarly, recent research in patients with coronary artery disease has identified multiple DNA methylation sites in blood leukocytes associated with adverse cardiovascular outcomes and mortality, highlighting the potential of DNA methylations as markers for prognosis and risk prediction (19). The strong discriminative performance observed in our study may support the potential clinical utility of global DNA methylation as a biomarker. However, this finding should be interpreted with caution. As the age difference between MI patients and controls, along with the use of healthy blood donors who may represent a “super healthy” population may lead to overestimation of the AUC, despite the adjustments and sensitivity analyses. Validation in larger and age-matched populations is therefore needed.

The observed association of MI with rDNA-CN and global DNA methylation may reflect parallel responses to genomic and epigenetic stress. MI is characterized by increased oxidative stress, inflammation, and cellular stress responses, which can disrupt genomic integrity and epigenetic regulation (30). Oxidative stress may induce DNA damage in repetitive regions such as rDNA (5), whereas inflammatory signaling and metabolic stress can alter the DNA methyltransferases activity, leading to global DNA methylation changes (19). In the literature, rDNA transcription is regulated by promoter methylation and nucleolar function (31); however, the relationship between rDNA-CN and global DNA methylation has not been fully characterized in this study. Further studies are warranted to clarify the causal relationship between MI and these molecular markers.

To our knowledge, this is the first study to investigate rDNA-CN in the context of MI and its relationship with global DNA methylation. Another strength of our study includes the use of ddPCR, which allows highly accurate and absolute quantification of rDNA-CN. However, several limitations should also be acknowledged. First, the relatively modest sample size may limit statistical power, particularly for detecting subtle associations such as those for rDNA-CN. Second, due to the cross-sectional design it is not possible to conclude whether the observed epigenetic alterations are a cause or consequence of MI. Systemic responses, such as inflammation and immune activation triggered by myocardial ischemia, may influence DNA methylation patterns in circulating leukocytes (32). Conversely, preexisting epigenetic dysregulation could potentially contribute to atherosclerosis progression and plaque instability (33, 34). Longitudinal studies with repeated measurements and genetic approaches, such as Mendelian randomization, are needed to clarify causality. Third, the use of peripheral blood as a surrogate for cardiac tissue is another limitation, as DNA methylation patterns can be tissue-specific and may not fully reflect changes in the myocardium or vascular wall. However, some overlap may arise because circulating leukocytes participate in inflammatory and immune processes underlying atherosclerosis and MI. They are exposed to systemic factors that influence epigenetic regulation across tissues. Consequently, blood-based methylation markers may capture systemic epigenetic alterations relevant to cardiovascular disease (19), although they may not represent tissue-specific changes. Finally, potential confounding factors beyond age and sex, such as unknown environmental exposures, could not be fully addressed.

## Conclusion

In summary, global DNA methylation was independently associated with MI and showed potential for distinguishing cases from controls, whereas rDNA-CN was not significantly associated with MI after adjustment for age and sex. These findings suggest that global DNA methylation may play a role in the pathogenesis of MI and could have potential as a biomarker. However, given the cross-sectional design and potential differences between the study and control group, further validation in larger and well-characterized populations is required.

## Data Availability

The original contributions presented in the study are included in the article/Supplementary Material, further inquiries can be directed to the corresponding author.

## Supplementary material

Supplementary Figure 1 **(A)** Correlation between rDNA-CN and age in all study participants. **(B)** Correlation between rDNA-CN and age stratified by patient group (Pearson's correlation).

Supplementary Figure 2 **(A)** Correlation between global DNA methylation and age in all study participants. **(B)** Correlation between global DNA methylation and age stratified by patient group (Spearman's correlation).

Supplementary Figure 3 **(A)** Correlation between rDNA-CN and global DNA methylation in all study participants. **(B)** Correlation between rDNA-CN and global DNA methylation stratified by patient group (Spearman's correlation).

Supplementary Figure 4 Schematic illustration of potential mechanisms linking MI with rDNA-CN and global DNA methylation. MI-related systemic stress responses, including oxidative stress, inflammation, and immune activation, may induce DNA damage and alter epigenetic regulation, affecting both rDNA stability and global DNA methylation. The downstream effects may contribute to atherosclerosis progression and plaque instability. The dashed “Unclear causality” box highlights the uncertainty about whether observed epigenetic alterations are causes or consequences of MI, emphasizing the need for longitudinal studies and Mendelian randomization.

Supplementary Table 1 Associations between rDNA-CN and characteristics of MI group (*n* = 100).

Supplementary Table 2 Associations between global DNA methylation and characteristics of MI group (*n* = 100)

## References

[1] Salim D Gerton JL. Ribosomal DNA instability and genome adaptability. Chromosome Res. (2019) 27(1):73–87. 10.1007/s10577-018-9599-7 30604343

[2] Rodriguez-Algarra F Evans DM Rakyan VK. Ribosomal DNA copy number variation associates with hematological profiles and renal function in the UK biobank. Cell Genomics. (2024) 4(6):100562. 10.1016/j.xgen.2024.100562 38749448 PMC11228893

[3] D'Aquila P Montesanto A Mandalà M Garasto S Mari V Corsonello A Methylation of the ribosomal rna gene promoter is associated with aging and age-related decline. Aging Cell. (2017) 16(5):966–75. 10.1111/acel.12603 28625020 PMC5595699

[4] Razzaq A Bejaoui Y Alam T Saad M El Hajj N. Ribosomal DNA copy number variation is coupled with DNA methylation changes at the 45s rdna locus. Epigenetics. (2023) 18(1):2229203. 10.1080/15592294.2023.2229203 37368968 PMC10305490

[5] Veiko NN Ershova ES Kondratyeva EI Porokhovnik LN Zinchenko RA Melyanovskaya YL Copy number variations of human ribosomal genes in health and disease: role and causes. Front Biosci (Landmark Ed). (2025) 30(2):25765. 10.31083/fbl25765 40018927

[6] Diesch J Hannan RD Sanij E. Perturbations at the ribosomal genes loci are at the centre of cellular dysfunction and human disease. Cell Biosci. (2014) 4(1):43. 10.1186/2045-3701-4-43 25949792 PMC4422213

[7] Kobayashi T. A new role of the rdna and nucleolus in the nucleus–rdna instability maintains genome integrity. Bioessays. (2008) 30(3):267–72. 10.1002/bies.20723 18293366

[8] Paredes S Maggert KA. Ribosomal DNA contributes to global chromatin regulation. Proc Natl Acad Sci USA. (2009) 106(42):17829–34. 10.1073/pnas.0906811106 19822756 PMC2764911

[9] Warburton PE Hasson D Guillem F Lescale C Jin X Abrusan G. Analysis of the largest tandemly repeated DNA families in the human genome. BMC Genomics. (2008) 9:533. 10.1186/1471-2164-9-533 18992157 PMC2588610

[10] Geisen ABC Santana Acevedo N Oshima J Dittrich M Potabattula R Haaf T. Rdna copy number variation and methylation during normal and premature aging. Aging Cell. (2025) 24:e14497. 10.1111/acel.14497 39853912 PMC12073889

[11] Law PP Mikheeva LA Rodriguez-Algarra F Asenius F Gregori M Seaborne RAE Ribosomal DNA copy number is associated with body mass in humans and other mammals. Nat Commun. (2024) 15(1):5006. 10.1038/s41467-024-49397-5 38866738 PMC11169392

[12] Valori V Tus K Laukaitis C Harris DT LeBeau L Maggert KA. Human rdna copy number is unstable in metastatic breast cancers. Epigenetics. (2020) 15(1-2):85–106. 10.1080/15592294.2019.1649930 31352858 PMC6961696

[13] Hosgood HD Hu W Rothman N Klugman M Weinstein SJ Virtamo JR Variation in ribosomal DNA copy number is associated with lung cancer risk in a prospective cohort study. Carcinogenesis. (2019) 40(8):975–8. 10.1093/carcin/bgz052 30859204 PMC6736087

[14] Chestkov IV Jestkova EM Ershova ES Golimbet VE Lezheiko TV Kolesina NY Abundance of ribosomal rna gene copies in the genomes of schizophrenia patients. Schizophr Res. (2018) 197:305–14. 10.1016/j.schres.2018.01.001 29336872

[15] Willmer T Johnson R Louw J Pheiffer C. Blood-Based DNA methylation biomarkers for type 2 diabetes: potential for clinical applications. Front Endocrinol (Lausanne). (2018) 9:744. 10.3389/fendo.2018.00744 30564199 PMC6288427

[16] Payne SR. From discovery to the clinic: the novel DNA methylation biomarker (M)Sept9 for the detection of colorectal cancer in blood. Epigenomics. (2010) 2(4):575–85. 10.2217/epi.10.35 22121975

[17] Kim M Long TI Arakawa K Wang R Yu MC Laird PW. DNA Methylation as a biomarker for cardiovascular disease risk. PLoS One. (2010) 5(3):e9692. 10.1371/journal.pone.0009692 20300621 PMC2837739

[18] Udali S Guarini P Moruzzi S Ruzzenente A Tammen SA Guglielmi A Global DNA methylation and hydroxymethylation differ in hepatocellular carcinoma and cholangiocarcinoma and relate to survival rate. Hepatology. (2015) 62(2):496–504. 10.1002/hep.27823 25833413

[19] Qin M Tian X Wu Q Zhu Q Yu M Fang X DNA Methylation predicts adverse outcomes of coronary artery disease. Nat Commun. (2025) 16(1):11396. 10.1038/s41467-025-66204-x 41387412 PMC12738899

[20] Jiao L Liu Y Yu X-Y Pan X Zhang Y Tu J Ribosome biogenesis in disease: new players and therapeutic targets. Signal Transduct Target Ther. (2023) 8(1):15. 10.1038/s41392-022-01285-4 36617563 PMC9826790

[21] Yan D Hua L. Nucleolar stress: friend or foe in cardiac function? Front Cardiovasc Med. (2022) 9:1045455. 10.3389/fcvm.2022.1045455 36386352 PMC9659567

[22] Memon AA Zöller B Hedelius A Wang X Stenman E Sundquist J Quantification of mitochondrial DNA copy number in suspected cancer patients by a well optimized ddpcr method. Biomol Detect Quantif. (2017) 13:32–9. 10.1016/j.bdq.2017.08.001 29021970 PMC5634817

[23] Vats S Sundquist K Wang X Zarrouk M Ågren-Witteschus S Sundquist J Associations of global DNA methylation and homocysteine levels with abdominal aortic aneurysm: a cohort study from a population-based screening program in Sweden. Int J Cardiol. (2020) 321:137–42. 10.1016/j.ijcard.2020.06.022 32593727

[24] Gutierrez JI Tyler JK. A mortality timer based on nucleolar size triggers nucleolar integrity loss and catastrophic genomic instability. Nat Aging. (2024) 4(12):1782–93. 10.1038/s43587-024-00754-5 39587368 PMC11964297

[25] Michler A Kießling S Durackova J Potabattula R Koparir A Haaf T. Rdna copy number variation and methylation from birth to sexual maturity. Aging (Albany NY). (2025) 17(6):1511–20. 10.18632/aging.206271 40527521 PMC12245198

[26] Kaimala S Yassin LK Hamad MIK Allouh MZ Sampath P AlKaabi J Epigenetic crossroads in metabolic and cardiovascular health: the role of DNA methylation in type 2 diabetes and cardiovascular diseases. Cardiovasc Diabetol. (2025) 24(1):231. 10.1186/s12933-025-02800-x 40442704 PMC12124063

[27] Dai Y Chen D Xu T. DNA Methylation aberrant in atherosclerosis. Front Pharmacol. (2022) 13:815977. 10.3389/fphar.2022.815977 35308237 PMC8927809

[28] Guo YQ Zhang JY Hou PP Jia CX Zhu TL Zhang QR Cross-Regulation of methylation and oxidative stress: molecular mechanisms and intervention strategies of diabetic cardiomyopathy. Eur J Pharmacol. (2025) 1005:178080. 10.1016/j.ejphar.2025.178080 40845960

[29] Navas-Acien A Domingo-Relloso A Subedi P Riffo-Campos AL Xia R Gomez L Blood DNA methylation and incident coronary heart disease: evidence from the strong heart study. JAMA Cardiology. (2021) 6(11):1237–46. 10.1001/jamacardio.2021.2704 34347013 PMC8340006

[30] Kibel A Lukinac AM Dambic V Juric I Selthofer-Relatic K. Oxidative stress in ischemic heart disease. Oxid Med Cell Longev. (2020) 2020(1):6627144. 10.1155/2020/6627144 33456670 PMC7785350

[31] Maehama T Nishio M Otani J Mak TW Suzuki A. Nucleolar stress: molecular mechanisms and related human diseases. Cancer Sci. (2023) 114(5):2078–86. 10.1111/cas.15755 36762786 PMC10154868

[32] Ward-Caviness CK Agha G Chen BH Pfeiffer L Wilson R Wolf P Analysis of repeated leukocyte DNA methylation assessments reveals persistent epigenetic alterations after an incident myocardial infarction. Clin Epigenetics. (2018) 10(1):161. 10.1186/s13148-018-0588-7 30587240 PMC6307146

[33] Lee HT Oh S Ro DH Yoo H Kwon YW. The key role of DNA methylation and histone acetylation in epigenetics of atherosclerosis. J Lipid Atheroscler. (2020) 9(3):419–34. 10.12997/jla.2020.9.3.419 33024734 PMC7521974

[34] Greißel A Culmes M Burgkart R Zimmermann A Eckstein HH Zernecke A Histone acetylation and methylation significantly change with severity of atherosclerosis in human carotid plaques. Cardiovasc Pathol. (2016) 25(2):79–86. 10.1016/j.carpath.2015.11.001 26764138

