# Supplementary material for: Associations of rDNA copy numbers and global DNA methylation with myocardial infarction
Source: Front Cardiovasc Med. 2026 May 4;13:1740048. doi: 10.3389/fcvm.2026.1740048 (PMC13180704; doi:10.3389/fcvm.2026.1740048)
Supplement: Supplementary Table 2 — Associations between global DNA methylation and characteristics of MI group (n = 100) [file Datasheet1.docx]

| **Table S1.** Associations between rDNA-CN and characteristics of MI group | | | | | | |
| --- | --- | --- | --- | --- | --- | --- |
|  | **Univariate** | | | **Adjusted for age and sex** | | |
| **Variables** | **β** | **P-value^a^** | **95% CI** | **β** | **P-value^a^** | **95% CI** |
| BMI | 2.83 | 0.05 | -0.04; 5.71 | 3.02 | 0.04 | 0.10; 5.94 |
| Smoking status (yes vs no) | 3.39 | 0.73 | -15.88; 22.66 | 1.55 | 0.88 | -18.90; 22.01 |
| Education (< 12 vs > 12 years) | -1.31 | 0.90 | -22.14; 19.51 | -2.01 | 0.85 | -23.78; 19.75 |
| Family history of MI (yes vs no) | -12.11 | 0.21 | -30.95; 6.73 | -12.73 | 0.21 | -32.94; 0.7.48 |
| Systolic blood pressure | -0.13 | 0.61 | -0.62; 0.37 | -0.15 | 0.65 | -0.70; 0.40 |
| Diastolic blood pressure | -0.16 | 0.73 | -1.07; 0.75 | -0.23 | 0.16 | -1.21; 0.76 |
| Triglycerides | 1.47 | 0.80 | -9.99; 12.94 | 1.50 | 0.80 | -10.44; 13.45 |
| Total cholesterol | -1.72 | 0.69 | -10.19; 6.74 | -3.38 | 0.46 | -12.45; 5.68 |
| HDL | 0.51 | 0.97 | -30.22; 31.23 | -5.41 | 0.75 | -38.43; 27.61 |
| LDL | 0.56 | 0.89 | -7.42; 8.54 | -0.26 | 0.95 | -8.52; 8.01 |

^a^Association tested by a linear regression model.

.

| **Table S2.** Associations between global DNA methylation and characteristics of MI group | | | | | | |
| --- | --- | --- | --- | --- | --- | --- |
|  | **Univariate** | | | **Adjusted for age and sex** | | |
| **Variables** | **β** | **P-value^a^** | **95% CI** | **β** | **P-value^a^** | **95% CI** |
|  |  |  |  |  |  |  |
|  |  |  |  |  |  |  |
| BMI | -0.002 | 0.35 | -0.01; 0.002 | -0.002 | 0.32 | -0.01; 0.002 |
| Smoking status (yes vs no) | 0.02 | 0.29 | -0.01; 0.05 | 0.02 | 0.24 | -0.01; 0.05 |
| Education (< 12 vs > 12 years) | 0.0003 | 0.98 | -0.03; 0.03 | -0.0002 | 0.99 | 0.03; 0.03 |
| Family history of MI (yes vs no) | 0.002 | 0.92 | -0.03; 0.03 | -0.0004 | 0.97 | -0.03; 0.03 |
| Systolic blood pressure | 0.001 | 0.07 | -0.0001; 0.001 | 0.001 | 0.18 | -0.001; 0.001 |
| Diastolic blood pressure | 0.001 | 0.25 | -0.001; 0.002 | 0.001 | 0.31 | -1.21; 0.76 |
| Triglycerides | -0.01 | 0.39 | -0.03; 0.01 | -0.01 | 0.39 | -0.03; 0.01 |
| Total cholesterol | -0.01 | 0.16 | -0.02; 0.004 | -0.01 | 0.19 | -0.02; 0.005 |
| HDL | 0.005 | 0.84 | -0.04; 0.05 | 0.01 | 0.69 | -0.04; 0.06 |
| LDL | -0.01 | 0.29 | -0.02; 0.01 | -0.01 | 0.34 | -0.02; 0.01 |

^a^Association tested by a linear regression model.


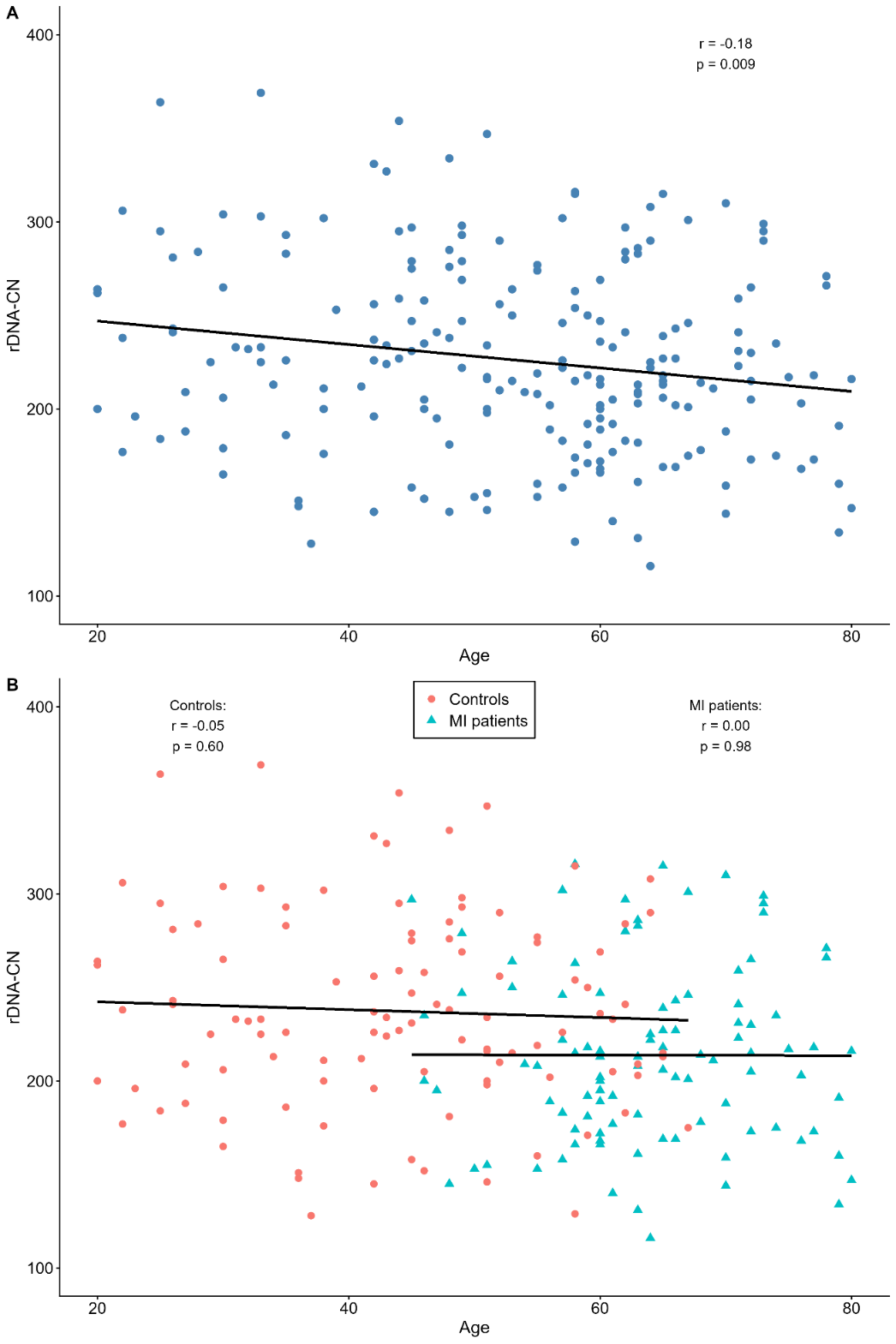


**Figure S1.** Correlation between rDNA-CN and age in all study participants, and within each group


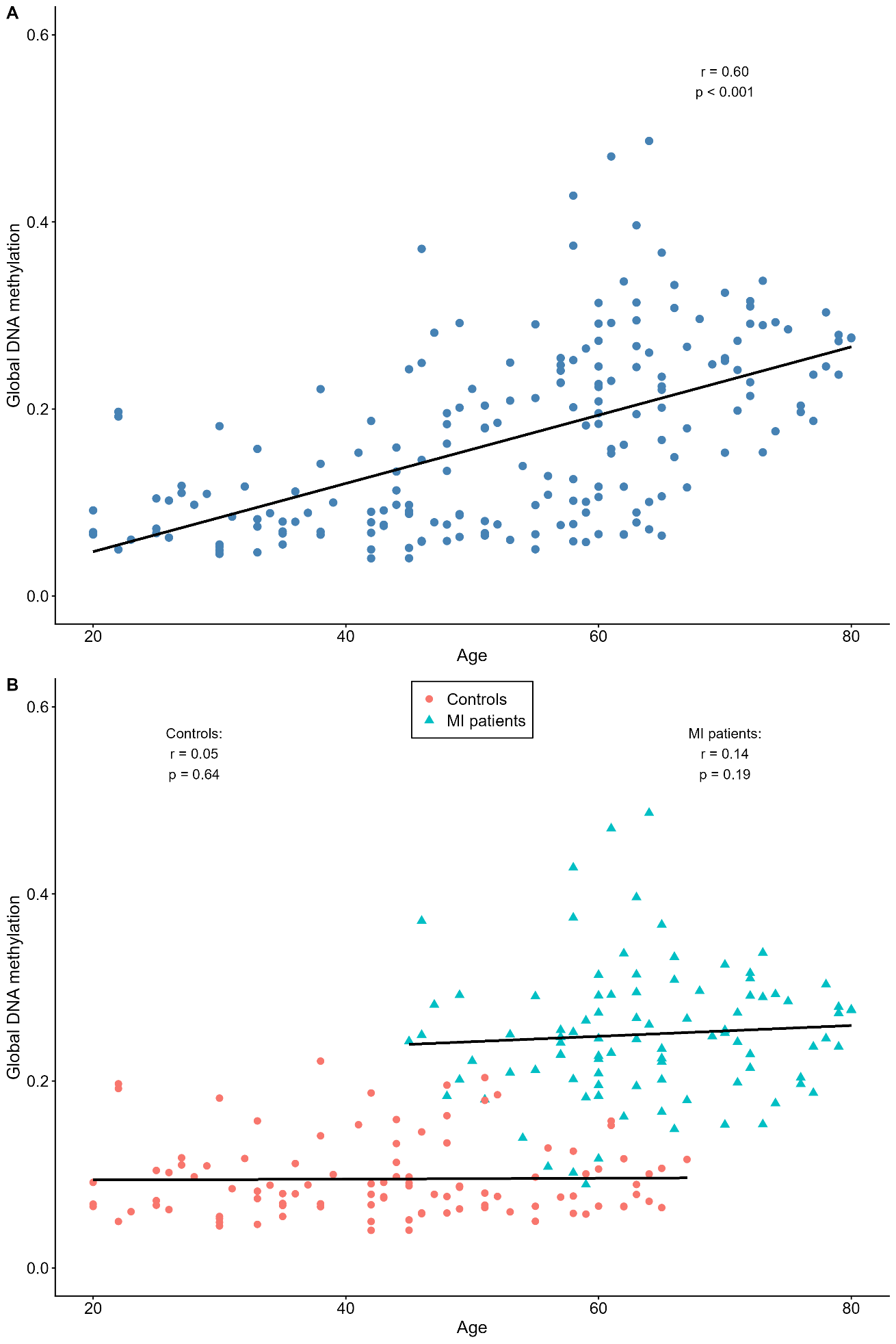


**Figure S2.** Correlation between global DNA methylation and age in all study participants, and within each group.

**
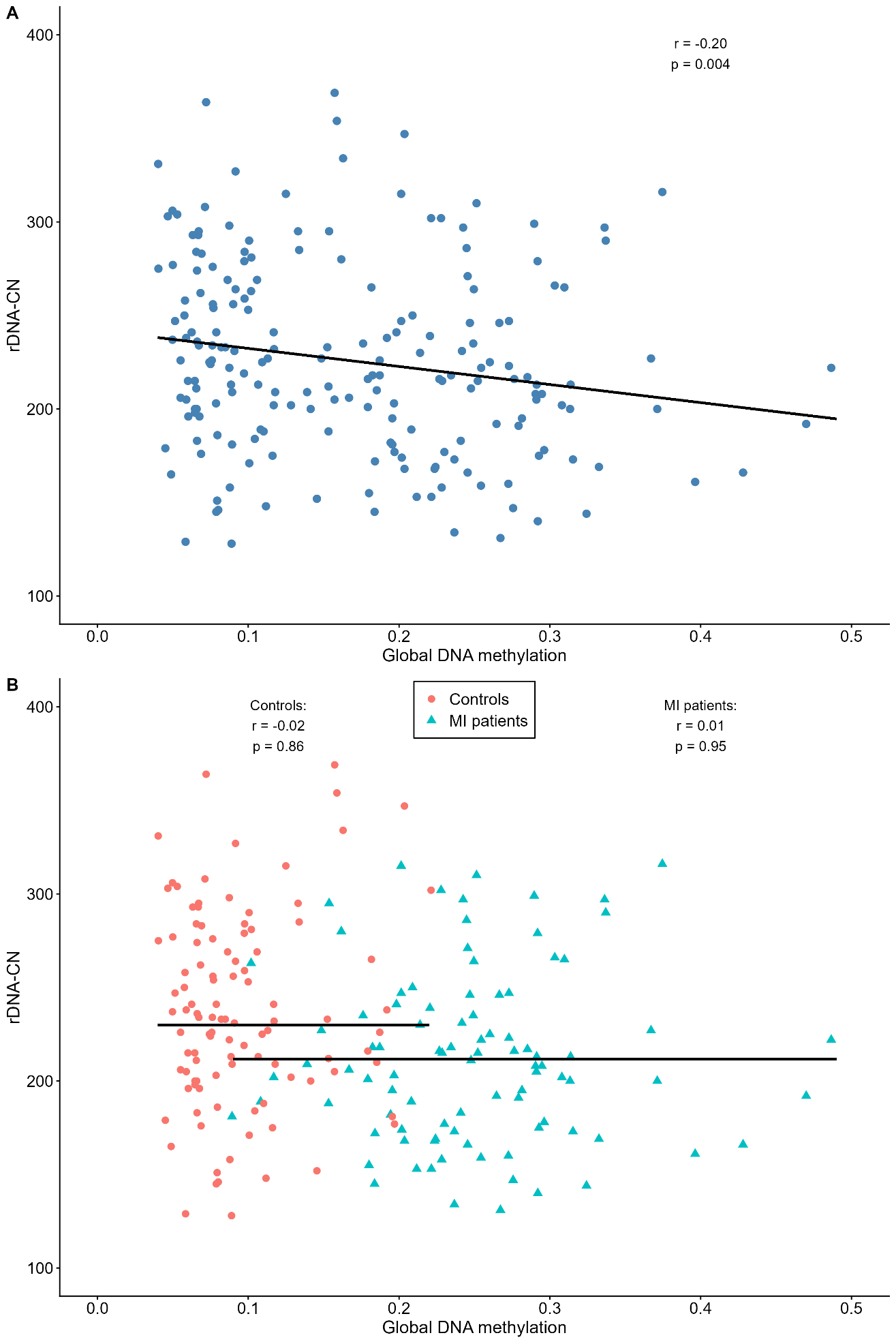
**

**Figure S3.** Correlation between rDNA-CN and global DNA methylation in all study participants, and within each group.


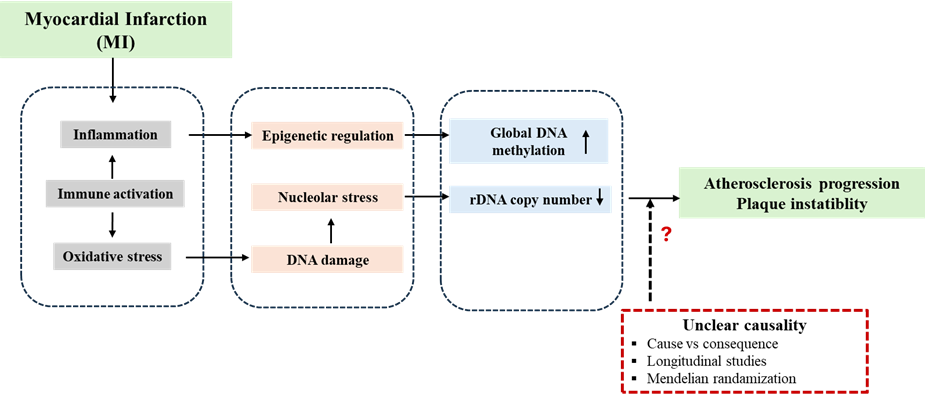


**Figure S4**. Schematic illustration of potential mechanisms linking MI with rDNA-CN and global DNA methylation. MI–related systemic stress responses, including oxidative stress, inflammation, and immune activation, may induce DNA damage and alter epigenetic regulation, affecting both rDNA stability and global DNA methylation. The downstream effects may contribute to atherosclerosis progression and plaque instability. The dashed “Unclear causality” box highlights the uncertainty about whether observed epigenetic alterations are causes or consequences of MI, emphasizing the need for longitudinal studies and Mendelian randomization.
